# Cardiac Rehabilitation—Current Paradigms and Challenges Across the Cardiovascular Continuum

**DOI:** 10.3390/jcm15166428

**Published:** 2026-08-20

**Authors:** Eduardo M. Vilela, Marta Catarina Almeida, Andreia Coelho, Sofia Viamonte, Amanda Pereira, Madalena Teixeira, Ricardo Fontes-Carvalho

**Affiliations:** 1 Cardiology Department, Unidade Local de Saúde de Gaia e Espinho, Rua Conceição Fernandes, 4434-502 Vila Nova de Gaia, Portugal; 2 Departamento de Medicina, Faculdade de Medicina, Universidade do Porto, 4200-319 Porto, Portugal; 3 Escola de Medicina, Universidade do Minho, 4710-057 Braga, Portugal; 4 Vascular Surgery Department, Unidade Local de Saúde de Gaia e Espinho, 4434-502 Vila Nova de Gaia, Portugal; 5 Unic@RISE, Faculdade de Medicina, Universidade do Porto, 4200-319 Porto, Portugal; 6 Centro de Reabilitação do Norte, Unidade Local de Saúde de Gaia e Espinho, 4405-565 Valadares, Portugal; 7 Diprofisio, 4100-343 Porto, Portugal

**Keywords:** cardiac rehabilitation, cardiology, cardiovascular prevention, preventive cardiology

## Abstract

Cardiac rehabilitation (CR) is a central component of optimized secondary prevention. Since their inception, CR programmes have progressively evolved. These incorporate several components in a multidisciplinary setting, aimed at addressing the diverse needs of the complex cardiovascular patient. From ischaemic heart disease to heart failure and cardio-oncology, CR has shown its relevance across various moments of the cardiovascular continuum. Notably, data has underscored the importance of an individualized assessment coupled to programme tailoring according to patient characteristics, to harness the full benefits of CR while ensuring safety. Against this background, current guidelines have endorsed CR in different clinical settings. Despite this, several challenges concerning CR implementation remain, including suboptimal referral and compliance rates, as well as underrepresentation of specific patient subgroups. In this article, we provide an overview concerning current paradigms for CR in contemporary clinical practice, while also exploring novel developments and future perspectives on this pivotal field.

## 1. Introduction

Over the years, several advances have profoundly transformed the management of cardiovascular disease (CVD) across the cardiovascular continuum [1,2,3]. From improvements in prevention strategies and cardiovascular risk factor (CVRF) management, to the advent of percutaneous coronary intervention, cardiac intensive care units, or novel paradigms in valvular heart disease, these have markedly changed perspectives concerning CVD [2,3,4,5]. Moreover, advances in understanding its pathophysiology, diagnostic modalities, and the interplay between CVD and conditions such as cancer or autoimmune diseases have also shaped current clinical practice [3,5].

While these have been instrumental in reducing morbidity and mortality, CVD is still a major burden [1,6,7,8,9]. Although its incidence is declining in regions such as Europe, CVD still represents the main cause of mortality worldwide, being a leading cause of morbidity and costs [1,7,8,9]. Global asymmetries and health inequities should also be recalled, as low- and middle-income countries account for a substantial proportion of deaths due to CVD [8].

Acknowledging differences in patient characteristics –across the lifespan, as well as between individuals—and incorporating patient-reported outcomes have gained growing recognition in modern healthcare [10,11,12,13,14]. Challenges posed by shifting demographics, CVRF trends, comorbidities, or social determinants of health have also progressively impacted care models [2,13,15,16,17]. This background has led to the need for comprehensive strategies to address the diverse specificities of patients with CVD, namely in secondary prevention settings [18,19,20]. Cardiac rehabilitation (CR) has emerged as a cornerstone of contemporary CVD management, incorporating several components in a structured programme, with data showing its interest in several clinical contexts [18,19,20,21,22,23].

In this narrative review, we provide an overview of some key indications of CR, as well as organizational aspects, workflows, and challenges to its optimal application. Furthermore, we also explore novel developments shaping contemporary CR landscapes such as cardio-oncology rehabilitation, peripheral artery disease (PAD), and its interplay with digital health.

## 2. Cardiac Rehabilitation and Personalization

### 2.1. From General Concepts to Individualized Care

**Figure 1:**
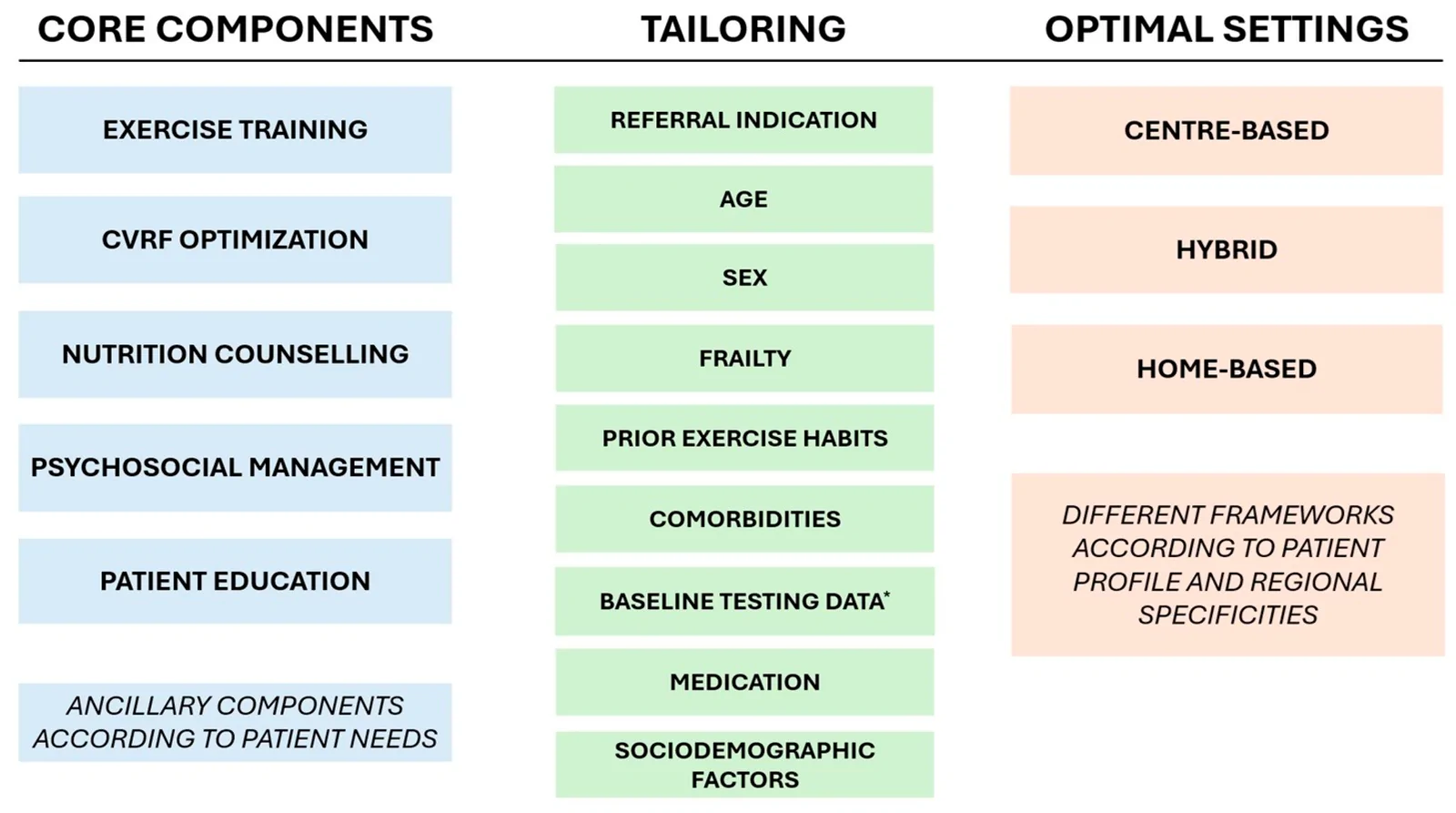
Cardiac rehabilitation—from general core concepts to personalized management. Contemporary cardiac rehabilitation (CR) programmes comprise a multidisciplinary structured intervention, encompassing several components. An individualized assessment and tailoring of programme components according to patient characteristics is a central part of CR. To tackle some of the barriers to CR implementation such as transportation difficulties, lack of CR centre coverage, or time constraints, frameworks such as hybrid or home-based programmes have also gained increased recognition (see main text for further details). CVRF, cardiovascular risk factors; *, including exercise stress testing, echocardiography and biochemical parameters.

CR programmes comprise comprehensive care workflows, encompassing several facets in a multidisciplinary setting (Figure 1) [18,19,20]. These include exercise training, CVRF management, nutrition counselling, psychosocial support, and ancillary components according to patient profiles [18,19,23,24,25]. A focus on patient education is also a pivotal part of these interventions [24,26,27]. Although exercise training constitutes a core part of CR, the integration of multiple complementary components is a key feature of these programmes [23,24,25,26].

Since its introduction, CR has profoundly evolved [28,29]. CR has continuously adapted in parallel to the overall management of CVD, progressing to address the needs of patients across time [19,28]. This can be illustrated by the marked changes in attitudes towards exercise training for patients with CVD, where highly restrictive views were progressively superseded by individualized risk stratification, recognizing the numerous benefits of exercise [30,31,32]. These include direct cardiovascular benefits—such as improvements in left ventricular and endothelial-derived relaxation—and positive effects on the musculoskeletal and autonomic nervous systems, as well as overall functional capacity [33,34,35,36]. Changes in CVRF management, including more stringent targets in arterial hypertension and dyslipidaemia, or the introduction of novel pharmacological therapies, have also influenced clinical approaches [20,37]. The recognition of the impact of mental health on cardiovascular health and disease has also raised awareness and reinforced the need for its assessment and appropriate management [38,39].

Particularities related to patient profiles—such as age and gender—have also gained the spotlight [19,40,41]. For example, whilst CR has shown its effectiveness in both women and the elderly, these groups are oftentimes less represented due to barriers such as lower referral and enrolment, while also presenting specific needs which should be accounted for when designing programmes [16,19,40,41,42,43,44]. Frailty is also a growing concern, as it affects a substantial number of patients and can influence clinical outcomes after CR [19,45,46]. At the opposite end of the spectrum, athletes and highly active individuals with CVD may also benefit from dedicated CR pathways [47,48]. In this context, coupling sports-specific assessments and return-to-play considerations may be particularly helpful [47,48].

### 2.2. Workflows and Organizational Issues

Although the general organizational framework and composition of CR programmes have previously been described in detail, it should be noted that specific members of the CR team perform different tasks under structured coordination [23,24,26]. The Secondary Prevention and Rehabilitation Section of the European Association of Preventive Cardiology (EAPC) of the European Society of Cardiology (ESC) has proposed minimal (mandatory for accreditation) and optimal (recommended) structure-based and process-based metrics, taking into consideration programme designs and settings [24]. Structure-based metrics encompass infrastructure (such as available facilities or equipment), human resources (including care-team composition and training), and centre requirements (such as protocols, strategic plans, or meeting schedules) [24]. Process-based metrics include the duration of the programme and the components of CR to be delivered [24]. These encompass a range of topics, namely including the types of testing—such as exercise stress testing or assessment of left ventricular function and arrhythmias—or cardiac monitoring which are available at CR centres [24]. The assessment of outcomes is also fundamental, with several indicators such as improvements in CVRF management, psychosocial status, or functional capacity having been described [24,26]. Moreover, quality indicators related to the number of potentially eligible individuals who enrol in and complete the predefined programme have also been proposed, allowing care teams to reflect on process optimization and refinements in patient care workflows [24,26].

While general standards for contemporary CR programmes have been reported by international scientific societies such as the EAPC, American Association of Cardiovascular and Pulmonary Rehabilitation, American Heart Association (AHA) and the American College of Cardiology (ACC), it should be acknowledged that specific frameworks have been proposed in different scenarios [24,26,49,50,51,52]. As detailed below, these aim to tackle the paradox between low referral rates and uptake, despite extensive data on the benefits of CR in distinct clinical contexts [49,51,53,54,55]. Both the advent of diverse technological aids and the recognition of numerous barriers to CR have led to renewed efforts to improve participation rates and reduce access inequities [50,51,55]. While the application of home-based or hybrid strategies may be useful, considering established standards of care and patient preferences is central to harness the full potential of these interventions while ensuring patient safety, particularly in higher risk subsets [50,51,55,56,57,58,59]. Furthermore, assessing different tools in specific groups of patients (such as the elderly or frail patients) is also pivotal to ensure that they offer added benefits when addressing these unmet needs [59,60].

## 3. Cardiac Rehabilitation Across the Cardiovascular Continuum

CR is a central component of CVD management [20,23,24,25,26,27,61,62]. This is reflected in contemporary guidelines, supporting its implementation across multiple clinical scenarios (Table 1) [20,27,63,64,65,66,67,68]. While data have classically focused on ischaemic heart disease (IHD), the relevance of CR in settings such as heart failure (HF) and cardio-oncology has garnered increasing attention [20,27,63,64,65,66,67,68,69,70]. Although CR has shown growing usefulness across a broad range of settings, factors such as patient characteristics, eligibility criteria, and programme designs must be considered when analyzing results [24,30,69,71].

### 3.1. Focus on Ischaemic Heart Disease

In IHD, CR is associated with reduced morbidity and mortality [21,74,75]. This is shown in a recent meta-analysis—including 85 randomized controlled trials—reporting its impact in reducing cardiovascular mortality, recurrent myocardial infarction, and hospitalizations [21]. These results are consistent with previous meta-analyses, reinforcing its application and relevance in this patient population [21,76]. Notably, the heterogeneity across studies should be underscored, as acknowledged in this analysis, which employed a random-effects model to account for this variability [21]. This concept has been previously discussed when addressing discrepancies among meta-analyses in this field, particularly concerning mortality outcomes [21,77]. Improvements in health-related quality of life (QoL) were also reported, as was the cost-effectiveness of CR [21]. Data also support the value of CR when focusing on contemporary settings, as reported in the Cardiac Rehabilitation Outcome Study (CROS) II meta-analysis encompassing randomized controlled trials as well as prospective and retrospective controlled cohort studies [74]. These findings are of special importance, attesting to the sustained role of CR, even when considering the numerous milestones in the management of IHD [19,20,21,27,29,63,64,74]. Indeed, the concept that CR programmes may present the most effective settings for secondary prevention strategies has strengthened their scope in contemporary clinical practice [19,20,27]. This is reflected in the class I, level A recommendation provided by both European and American guidelines for CR (both for acute and chronic coronary syndromes) [20,27,63,64,65].

Importantly, as mentioned above, heterogeneity across studies should be acknowledged [21,69,71,74,75,76,77,78,79,80]. When analyzing different studies, it should be kept in mind that programme designs, patient features, and the specific clinical setting (namely acute versus chronic coronary syndromes, or the type of interventions performed) may influence results [21,69,71,74,75,76,77,78,79]. Delays in CR initiation can also hinder its efficacy, as these may lead to additional time needed to accrue benefits [30]. This point is reflected in the quality indicators proposed for CR, which incorporate the time elapsed from referral to programme initiation—ideally within 14 days for myocardial infarction and 28 days for coronary artery bypass grafting (this is potentially earlier in experienced centres, while accounting for healing issues post-surgery) [24]. Subgroups such as patients who suffered an acute coronary syndrome due to a spontaneous coronary artery dissection (SCAD) may also present specific challenges, namely due to patient age or safety considerations [80]. In this regard, despite limitations such as its retrospective design, relatively small number of patients, and lack of long-term assessment, the Modalities and Safety of Cardiac Rehabilitation in a Population Managed for Spontaneous Haematoma or Coronary Disruption (READAPT-DISCO) study provided encouraging data on the feasibility of CR in SCAD survivors [80].

Another issue which should be considered pertains to exercise prescription [30,31,69,81,82,83]. As for pharmacological therapy, and also in exercise training, the “dose” may influence results [84,85,86]. Indeed, the FITT(VP) framework for exercise prescription—encompassing frequency, intensity, time, type, volume, and progression—shares parallels with the dose-exposure-response model of pharmacological interventions [87]. While a detailed account of exercise prescription in CVD is beyond the scope of this article, recalling some core concepts is fundamental when analyzing the literature [30,31,83,86]. Both aerobic and resistance training should be considered, while the patient’s comorbidities (among which musculoskeletal conditions or limitations, and history of stroke) must be accounted for [19,23]. Performing a baseline assessment of the individual’s functional capacity has implications for both prescription (namely in terms of exercise intensity) and prognosis [30,31,83]. Methodologies such as cardiopulmonary exercise testing (CPET) provide objective information in both domains, being highly useful in CR [24,30,31,83,88,89,90,91,92]. CPET provides an ideal framework for these assessments, with peak oxygen consumption (VO_2peak_) being the gold standard for the assessment of functional capacity, while ventilatory thresholds are considered the reference standard for aerobic exercise prescription [31,83]. While submaximal tests—such as the six-minute walk test (6MWT)—can be of interest in some settings (namely when addressing walking ability), they present substantial limitations when compared to CPET or exercise stress testing with electrocardiographic monitoring [24,83]. As stated in an EAPC position statement, the 6MWT is not adequate for exercise risk stratification [24,83]. Importantly, despite some caveats, functional capacity has been linked to outcomes, with greater improvements after CR being associated with event reductions [88,89,90,91,93]. Notably, improvements in VO_2peak_—when assessed as a continuous variable—have been linked to lower mortality [88,91,92]. An analysis of 1171 coronary artery disease patients undergoing CR comparing those without improvements in VO_2peak_ to those with a low (<2.5 mL/kg/min) or high (≥2.5 mL/kg/min) response also showed a significantly higher mortality in those with no or low improvements, as compared to those with a high response [91]. Interestingly, attendance to CR has also been associated with outcomes, a fact which may in turn be related (at least in part) to functional improvements [94,95]. Nevertheless, factors such as increases in medication persistence in those undergoing CR should also be considered when assessing overall patient benefits [62]. Both are also incorporated as quality indicators for CR, once more reinforcing the need for an integrated patient evaluation throughout the programme [24]. As mentioned above, CVRF optimization—including the uptitration of antidyslipidaemic or antidiabetic medication and continuous adjustments to antihypertensive drugs according to patient needs—is central to CR [20,24,26]. Finally, as echoed in an EAPC consensus statement, a detailed assessment of possible barriers to medication adherence, including psychosocial issues, should also be performed [96]. The multidisciplinary nature of CR can be key to overcoming these hurdles and promoting long-term compliance with guideline-directed medical therapy [37,96].

### 3.2. Heart Failure

While maintaining a pivotal role in IHD, CR has also become central in other entities [19,20,70,97,98]. Among these, several studies have addressed the role of CR in HF [22,25,99,100,101]. A contemporary Cochrane systematic review and meta-analysis of randomized trials—using a random-effects model—showed the benefits of CR in reducing hospitalizations and in significantly improving health-related QoL [22]. As for IHD, the heterogeneity among HF studies should be taken into consideration [22,98,100,101]. This is depicted in different meta-analyses, yielding distinct results (namely in terms of hospitalizations) [22,100]. When addressing this matter, several points should be considered [22,98,100,101,102,103].

First, as discussed in the guidelines, programme adherence may affect results [66]. In addition, programme structure, compliance, and response status might also influence outcomes [22,42,66,104,105]. An analysis from the Heart Failure: A Controlled Trial Investigating Outcomes of Exercise Training (HF-ACTION) multicentre randomized controlled trial showed that distinct patterns of functional capacity (measured via VO_2peak_ derived from CPET) were associated with differential clinical outcomes [104]. Specifically, each 6% increase in VO_2peak_ from baseline to three months was associated with a 5% reduction in the risk of a composite endpoint encompassing all-cause mortality or hospitalization [104]. While these data should be interpreted considering the lack of significant differences in the composite primary endpoint (all-cause mortality or hospitalization) in this seminal trial comparing usual care plus aerobic exercise to usual care alone in HF patients, they nonetheless provided valuable insights [99,104]. Of note, the magnitude of functional improvements in this study (a median of 4% increase in VO_2peak_ from baseline) was relatively small when compared to other studies, whereas compliance issues were also prevalent [99,104,105]. In addition, programme duration may also influence overall benefits [99,106]. Second, given the multisystem impact of HF, an integrative workflow should be considered [19,97,105,107]. In exercise training, both aerobic and resistance training are essential, whereas some data also show potential benefits for ancillary methodologies such as inspiratory muscle training [19,31,107,108,109]. As discussed above, ventilatory thresholds (measured by CPET) provide ideal ranges for aerobic training, allowing a more precise prescription when compared to methods based on heart rate percentages or submaximal data [24,31,83,107]. For resistance training, assessing the one-repetition maximum and using the adult Omnibus Resistance Exercise Scale (OMNI-RES) allows for the tailoring of this component [31]. Although the safety of resistance training in clinically stable HF patients has been reported, factors such as the presence of a cardiac implantable electronic device (CIED), blood pressure levels or diabetes (namely in the case of retinopathy) should be assessed, to allow adequate regimens [31,109]. A dedicated and multiparametric assessment may provide additional information, which might allow further patient phenotyping and personalized management [31,110,111]. CPET may be particularly useful in providing information on various phenomena which can affect HF patients, across a range of ejection fractions [30,92,110,112]. Beyond usual CPET protocols, incorporating invasive measurements (invasive CPET) and its integration with echocardiography (CPET-echo) have also been described to further refine patient assessment [110,111,112]. Finally, the significant improvements in functional capacity and QoL shown after CR should be highlighted [22,98,100,112]. These have been consistent across analyses, providing pertinent insights into this topic [22,97,98,100,113].

Although HF with a reduced ejection fraction has predominated in the literature, studies have also explored the role of CR in HF with preserved ejection fraction (HFpEF) [20,22,97,98,114]. It should not be overlooked that this syndrome affects multiple systems, with exercise training being a central intervention to counteract its adverse impact [97,110,115]. Beyond exercise training, advances in the understanding of HFpEF and its therapeutic options have markedly changed its management in recent years, with the introduction of sodium-glucose cotransporter 2 inhibitors, nonsteroidal mineralocorticoid receptor antagonists, and incretin-based therapies [116]. Importantly, CR has been associated with improvements in functional capacity, QoL, and symptomatic burden among HFpEF patients [114,117,118]. Despite this, as highlighted in different meta-analyses—applying random-effects models and focusing on randomized clinical trials—in this field, data assessment remains hampered by heterogeneity across studies. This includes discrepancies in patient definitions (such as including patients within the spectrum of reduced ejection fraction), programme structure, or QoL assessment methodologies [22,114]. Moreover, as stated in a recent ACC expert consensus document, challenges related to patient participation (including in terms of reimbursement) may also further limit their representation [116]. Nonetheless, while recognizing the need for large multicentric studies concerning HFpEF, CR may be useful in this challenging clinical context, particularly in those with frailty, comorbidities, and more severe disease phenotypes [19,22,66,117,118].

CR programmes also provide prime settings for guideline-directed medical therapy optimization, as well as reinforcing patient education and the importance of medication adherence [119]. Diuretic adjustment and medication reconciliation (including noncardiovascular drugs) are also important points to be addressed throughout the programme [119]. Notably, as detailed above, different CR components (such as exercise training, medication titration, or psychosocial management) should be integrated to ensure an optimal approach to the individual patient [18,19,98,119].

Given the toll HF can have on patient autonomy and QoL, and the burden presented by symptoms such as dyspnoea and fatigue, these aspects reinforce the relevance of CR in this patient population [22,97,98]. This concept is echoed by current guidance documents; HF guidelines provide a class IIa recommendation for CR (level B according to the ACC/AHA and level C from the ESC), whereas the ESC guidelines on CVD prevention in clinical practice provide a class I, level A recommendation [20,66,67].

### 3.3. Cardiac Electronic Implantable Devices and Advanced Heart Failure

Studies have also consistently shown the safety of CR in HF, including in patients with more advanced disease phenotypes and in the presence of a CIED [97,98,120,121,122]. When evaluating individuals with a CIED, beyond standard assessments, a dedicated analysis of the device (both at rest and during exercise) is needed to allow adequate exercise prescription while ensuring safety and providing patient reassurance [122]. The underlying CVD is pivotal to an individualized approach concerning different programme components, while CIED-specific issues (including type of device, programming parameters, and therapy zones in the case of defibrillators) must also be considered [30,109,122]. The presence of arrhythmias—both ventricular and supraventricular, which can lead to inappropriate shocks in the case of defibrillators—must also be addressed [122]. These may require further therapeutic adjustments, including in terms of the exercise programme [122]. Tailored recommendations for physical activity and sports counselling, encompassing these domains, should also be implemented [30,122].

In heart transplant settings, CR can also be a relevant component [19,123]. This multidisciplinary intervention is particularly suited for this scenario, where several factors—ranging from the surgical procedure to immunosuppression, psychosocial burden, and skeletal muscle dysfunction—may hamper patient recovery, functional capacity, and QoL [123,124,125]. This is reflected in the guidelines from the International Society for Heart and Lung Transplantation (ISHLT), which provide a class I, level B recommendation for CR in those after a heart transplant [72]. According to timing after transplantation (early after the procedure versus post-discharge), CR should be tailored to account for specific issues—at different timepoints—such as wound healing and clinical stabilization, avoidance of environmental risks, or potential rejection [123]. Although different training methods may be applied, it should be underscored that heart rate monitoring is limited in the face of denervation-related abnormal chronotropic responses [123]. Particularly early on, a warm-up period before the exercise session is relevant in this context, as detailed in a clinical consensus on rehabilitation after cardiac transplantation [123].

Furthermore, the ISHLT guidelines also provide a class IIa, level C recommendation for CR in those awaiting heart transplantation to reduce readmissions, mortality whilst on the wait list, and to improve post-transplant outcomes [72]. While, as reflected in the level of recommendation, the role of CR in the pre-transplant period to optimize outcomes is still not fully ascertained, these data once more reinforce the transversal nature of CR along the patient journey [72,126].

In those with a left ventricular assist device (LVAD), exercise-based CR may provide benefits such as improvements in functional capacity and QoL [19,127,128,129]. Despite some data on this issue, and the rationale for its application, optimal designs concerning exercise training and CR in LVAD recipients are still not fully ascertained [19,127,128,129,130,131].

### 3.4. Cardio-Oncology and Beyond

Cardio-oncology is another rapidly expanding area where CR can be beneficial [68,70,132]. The importance of exercise training across the cancer-patient journey has been strongly endorsed in diverse settings, while the prognostic relevance of functional capacity has also been highlighted [30,68,133,134,135,136,137,138]. Regarding the former, incorporating exercise training in cancer care can offer several benefits, such as improvements in functional capacity and QoL [30,68,133,134,136,137,138,139,140]. As for the latter, the role of functional capacity is elegantly illustrated by its use in settings such as non-small cell lung cancer, where VO_2peak_ can assist in risk stratification when considering surgical treatment [140].

Data has also emerged showing that beyond its role as a supportive measure, exercise could have a direct impact on cancer physiopathology [141,142,143]. This has been described in both mechanistic and animal studies, as well as in human diseases such as breast and prostate cancer [141,142,143,144,145]. Several limitations should, however, be noted. These include relatively small patient cohorts, variations in tumour type and staging, different study designs, and potential safety considerations. Nonetheless, these studies provide valuable data concerning the role of exercise training in this field [141,142,143,144,145,146]. More recently, a randomized controlled trial on patients with colon cancer who underwent adjuvant chemotherapy after resection provided additional insights [147]. In this study, patients were randomized to exercise training or health education material alone, with those assigned to exercise presenting significantly longer disease-free survival (the study’s primary endpoint) [147]. Interestingly, overall survival was also longer in the exercise group [147]. Although, as discussed by the authors, limitations such as recruitment issues, the number of events, or initial health status should be acknowledged, these findings further reinforce the case for exercise oncology [147]. This is reiterated in current guidelines, recommending exercise training across different moments of the cancer-patient journey [30,68,133,134,135,136].

Beyond exercise training, dedicated CR programmes can provide additional benefits in selected individuals, such as cancer survivors at high cardiovascular risk [68,70]. Given the complex bidirectional relationship between CVD and cancer, including possible cardiotoxicity from several anticancer treatments as well as the various common pathways and risk factors, cardio-oncology rehabilitation (CORE) has emerged as an important intervention [39,68,70,132,148]. These programmes include traditional CR tenets, while also incorporating aspects specifically related to cancer management [70,132,149]. In this context, a study showed that, compared to usual care (including community-based exercise training), CORE was associated with significantly greater improvements in functional capacity, health-related QoL, risk factor control, and health literacy among cancer survivors with high cardiovascular risk [148]. Data also show that, when compared to community-based exercise training, CORE may be associated with greater compliance rates and patient satisfaction, while remaining cost-effective [150,151]. Notably, consideration should be given to generalization issues—such as the single-centre nature of this study and the specific cancer types investigated (mostly breast cancer and lymphoma)—as well as the need for data from larger multicentric randomized controlled trials [148]. While, as acknowledged in a whitepaper by the International Cardio-Oncology Society, several hindrances persist concerning the optimal application of CORE, current data nonetheless support its clinical utility in selected patient groups [70,152,153]. These have been endorsed by the cardio-oncology guidelines from the ESC, which provide a class IIa, level A recommendation for dedicated CR among cancer survivors at high cardiovascular risk [68].

Finally, exercise training has emerged as a core component in the management of PAD [73,154,155,156]. Supervised exercise training improves walking distance, pain-free walking distance, and QoL, being a safe intervention [73,154,155]. As for conditions such as IHD and HF, an adequate and personalized exercise prescription is pivotal to maximize its benefits [154,155,157,158]. This is endorsed in current European and American guidelines, which provide a class I, level A recommendation for supervised exercise training in symptomatic PAD [73,155,156]. Interestingly, as described in American guidelines, CR programmes may be ideal settings for this provision [156]. Given cardiovascular risk profiles, potential gains from other CR components (beyond exercise training) could be particularly relevant [19,159,160]. Moreover, some data supports the application of CR in this patient population [161]. Despite this, and as highlighted in the guidelines, further prospective randomized data on CR—particularly compared with usual care, including supervised exercise training alone—are warranted to define the optimal application of this intervention in PAD [73,162].

Valvular heart disease is another area where the role of CR has evolved [19,25,163]. Although studies have reported benefits in functional capacity, QoL, and cost-effectiveness, it should be recognized that large-scale randomized controlled trial data is limited in this scenario [19,25,163]. Interestingly, in recent years data has also emerged on the potential role of prehabilitation to optimize postprocedural recovery [164,165]. This has been shown in a multicentre randomized controlled trial where patients allocated to teleprehabilitation had a significantly lower rate of hospitalizations (a component of the study’s primary endpoint) [164]. A meta-analysis of randomized controlled trials (including four studies) also supported the potential utility of prehabilitation, though underscoring substantial heterogeneity and risk of bias across studies [165]. Notably, and given the overlap with HF, addressing the overall role of CR in individuals undergoing percutaneous interventional procedures (such as mitral valve intervention) is also a growing field [163]. As for those undergoing transcatheter aortic valve implantation (TAVI), there is also encouraging data in terms of functional capacity and QoL, with a retrospective cohort study in England reporting on outcome benefits (though, as discussed by the authors, this is limited due to the study design and potential confounding factors) [166,167].

## 4. Challenges to CR—Barriers, Unmet Needs, and Future Perspectives

Despite having far-reaching applications, and being strongly endorsed by contemporary guidelines, various reports underscore the suboptimal implementation of CR [20,23,27,53,54,55]. Data from Europe, derived from the ESC EURObservational Research Programme (EORP) European Action on Secondary and Primary Prevention by Intervention to Reduce Events (EUROASPIRE) V registry, indicate that fewer than half of patients with coronary artery disease are advised to participate in CR programmes [168]. A survey across ESC member countries (where 42 out of the 51 potential countries provided valid responses) showed that in more than half of the surveyed countries, CR uptake after a myocardial infarction was ≤50% [53]. The multicentric International Action on Secondary Prevention through Intervention to Reduce Events (INTERASPIRE) study reinforced this landscape, showing substantial gaps in CR referral and attendance, as well as marked regional variation [169,170]. Other studies have showed similar patterns, with CR referral and subsequent adherence below ideal [54,55,171].

Several barriers have been identified as potential factors leading to these results [55,171]. As elegantly described by Taylor et al., these encompass various levels, including patient, programme, clinician and healthcare/system-level factors [55]. While acknowledging variations between regions, some patient subgroups—such as women, the elderly, or socioeconomically disadvantaged patients—may be particularly underrepresented [40,172,173]. Notably, studies have demonstrated the benefits of CR in these subgroups, further strengthening the need for strategies to improve CR implementation [40,41,42,174,175,176]. Healthcare system economics should also be considered, as issues such as high out-of-pocket costs, lack of funding (potentially affecting the availability of CR facilities or dedicated equipment), or reimbursement difficulties may also affect CR implementation [55,171,177].

To address the multidimensional nature of the barriers to CR, proposed solutions also encompass various levels [55,171]. These include strategies ranging from increasing awareness to CR (in both patients and clinicians) to reinforcing training of specialized CR staff and expanding coverage across regions [55,161,178,179]. In terms of accessibility, the advent of home-based and hybrid frameworks has also been hailed as a potential strategy in some individuals [23,50,51,52,55,56,57]. These could be particularly useful to overcome transportation hurdles and geographical disparities, while also potentially mitigating factors such as time constraints [55,171]. Although differences related to populations under study, geographical location, or programme structure should be noted, digital platforms may provide cost-effective alternatives in some settings [180,181]. Despite having been described for some time, the disruptive COVID-19 (coronavirus disease 2019) pandemic has led to a renewed interest in these models, while adding further insights into their application [58,182]. Despite the potential usefulness of these interventions, tailoring them to specific settings and individual patient needs is essential when considering their application [50,51,56,182].

Finally, novel technological advancements continue to shape cardiovascular care and to offer diverse opportunities within CR [183,184,185,186]. Some examples include the use of remote monitoring to assess metrics such as heart rate during CR sessions. Additionally, data derived from various wearables—encompassing this and other parameters such as weight, glucose levels, and step counts—may also be informative when optimizing secondary prevention strategies [184,185]. While acknowledging improvements, the unmet need concerning CVRF goal achievement even among those undergoing CR also reinforces the interest in possible ancillary strategies to improve overall secondary prevention measures [170,175,187,188,189].

Artificial intelligence (AI) also continuously challenges previous paradigms concerning CVD management [184,186,190]. While a relatively novel field when considered in this context, the application of AI (across its various types and iterations) could also provide ample opportunities for streamlining CR [184,190,191,192,193]. These may include data processing from different sources (namely including wearables, as mentioned above) and pattern recognition, and assisting adherence as well as programme fine tuning [184,190,191,192,193]. While the optimal application of these workflows is still not fully ascertained, the rapid progression of AI models across different areas is potentially set to also have a substantial impact on CR [184,190,191,192,193].

## 5. Conclusions

CR provides a comprehensive framework designed to meet the multifaceted needs of the complex cardiovascular patient. This time-tested intervention has markedly progressed since its inception, incorporating components from various areas while evolving to face current challenges. While its benefits in settings such as IHD, HF, or cardio-oncology have been shown in different studies, suboptimal CR implementation presents a substantial limitation to its application. As cardiovascular care continues to progress towards increasingly personalized and technology-enabled models, CR remains at the forefront of optimal secondary prevention, uniquely positioned to integrate evidence-based interventions to improve outcomes, QoL, and long-term cardiovascular health.

## Figures and Tables

**Table 1:** Overview of some of the contemporary guidance concerning cardiac rehabilitation. Cardiac rehabilitation is a multidimensional intervention encompassing exercise, cardiovascular risk factor management, nutritional counselling, and psychosocial support. Patient education, alongside optimization of guideline-directed medical therapy or ancillary components according to patient needs, is also part of these programmes (see main text for details).

	Class of Recommendation/Level of Evidence	Scientific Society
Acute coronary syndromes	I/A	ESC [20,27]
	I/A	ACC/AHA/ACEP/NAEMSP/SCAI [64]
Chronic coronary syndromes	I/A IIa/B for HBCR	ESC [20,63]
	I/A (after recent MI, PCI, or CABG) I/B-R (with stable angina or after heart transplant) I/C-LD (after recent SCAD event)	AHA/ACC/ACCP/ASPC/NLA/PCNA [65]
Heart failure	I/A versus IIa/C *	ESC [20,66]
	IIa/B-NR	AHA/ACC/HFSA [67]
Heart transplant	I/B I/B for HBCR or HCR as potential alternatives to centre-based cardiac rehabilitation IIa/C for patients awaiting heart transplantation	ISHLT [72]
Cardio-oncology	IIa/B	ESC/EHA/ESTRO/IC-OS [68]
Peripheral artery disease	IIb/B	ESVS [73]

ACC = American College of Cardiology; ACCP = American College of Clinical Pharmacy; ACEP = American College of Emergency Physicians; AHA = American Heart Association; ASPC = American Society for Preventive Cardiology; CABG = coronary artery bypass grafting; EHA = European Hematology Association; ESC = European Society of Cardiology; ESTRO = European Society for Therapeutic Radiology and Oncology; ESVS = European Society for Vascular Surgery; HBCR = home-based cardiac rehabilitation; HCR = hybrid cardiac rehabilitation; HFSA = Heart Failure Society of America; IC-OS = International Cardio-Oncology Society; ISHLT = International Society for Heart and Lung Transplantation; MI = myocardial infarction; NAEMSP = National Association of EMS Physicians; NLA = National Lipid Association; PCI = percutaneous coronary intervention; PCNA = Preventive Cardiology Nurses Association; SCAD = spontaneous coronary artery dissection; SCAI = Society for Cardiovascular Angiography and Interventions. * I/A in the 2021 ESC guidelines on cardiovascular disease prevention in clinical practice; IIa/C in the 2021 ESC Guidelines for the diagnosis and treatment of acute and chronic heart failure.

## Data Availability

No new data were created or analyzed in this study. Data sharing is not applicable to this article.

## Abbreviations

The following abbreviations are used in this manuscript:

6MWT	Six-minute walk test
ACC	American College of Cardiology
AHA	American Heart Association
AI	Artificial intelligence
CIED	Cardiac implantable electronic device
CORE	Cardio-oncology rehabilitation
COVID-19	Coronavirus disease 2019
CPET	Cardiopulmonary exercise testing
CVD	Cardiovascular disease
CVRF	Cardiovascular risk factor
CR	Cardiac rehabilitation
EAPC	European Association of Preventive Cardiology
ESC	European Society of Cardiology
HF	Heart failure
HFpEF	Heart failure with preserved ejection fraction
IHD	Ischaemic heart disease
ISHLT	International Society for Heart and Lung Transplantation
LVAD	Left ventricular assist device
PAD	Peripheral artery disease
QoL	Quality of life
SCAD	Spontaneous coronary artery dissection
VO_2peak_	Peak oxygen consumption
